# Supplementary material for: Delineating Mycobacterium abscessus population structure and transmission employing high-resolution core genome multilocus sequence typing
Source: Nat Commun. 2022 Aug 23;13:4936. doi: 10.1038/s41467-022-32122-5 (PMC9399081; doi:10.1038/s41467-022-32122-5)
Supplement: Supplementary file 1 — Supplementary Information [file 41467_2022_32122_MOESM1_ESM.pdf]

# **Delineating *Mycobacterium abscessus* population structure and transmission employing high-resolution core genome multilocus sequence typing**

Margo Diricks, Matthias Merker, Nils Wetzstein, Thomas A. Kohl, Stefan Niemann, Florian P. Maurer

# Supplementary Methods

## 1. Creation of *Mycobacterium abscessus* cgMLST scheme

Core loci were defined using the cgMLST target definer v1.5 implemented in the SeqSphere+ software (client v7.7.5). The finished genome of the *Mycobacterium abscessus* subsp. *abscessus* ($Mab_A$) ATCC19977 type strain was used as seed genome. All finished genome assemblies (chromosome or complete genome level) available on July 2$^{nd}$, 2021 (n=67) as well as 31 draft genomes (contig or scaffold level) were considered for scheme creation (n=98). The draft genomes were added to better span the entire diversity of Mab (Supplementary Fig. 1) and, in particular, to provide representatives for all seven previously described dominant circulating clones (DCC[1]). More specifically, no finished genomes were available for DCC5 and DCC6, leaving only draft genomes as alternative. In addition, the use of draft genomes can be useful to eliminate loci from the scheme which are hard to assemble (e.g. loci with large repetitive regions). An initial quality screen with Ridom SeqSphere+ (v7.7.5) software identified two outliers (NZ_CP065033.1 and NZ_CP022230.1; finished genomes; both subspecies *massiliense*) with more than 1,000 missing/bad cgMLST targets, which were therefore removed. The final scheme creation set thus comprised one seed genome (ATCC19977) and 96 penetration query genomes (Supplementary Data 1) including 58 $Mab_A$, 30 $Mab_M$ and 8 $Mab_B$ with representatives for all seven DCCs and from at least 11 countries (South Korea, Russia, USA, China, Malaysia, UK, Canada, France, Spain, Australia and Netherlands) (Supplementary Fig. 1). All publicly available plasmids (n=17 on July 2$^{nd}$, 2021; CU458745.1, NC_021278.1, NZ_AP022622.1, NZ_CP065264.1 NZ_CP065184.1, NZ_CP063329.1, NZ_CP065285.1, NZ_CP063325.1, NZ_CP063321.1, NZ_CP065281.1, NZ_CP065277.1, NZ_CP065270.1, NZ_CP065267.1, NZ_CP022234.1, NZ_CP050977.1, NZ_ATFQ01000044.1, NC_020994.1) were used to exclude plasmid-borne sequences from the scheme.

As first step of the scheme creation process, all protein-coding genes were extracted from the seed genome (n=4,920) and filtered according to the default SeqSphere+ Seed Genome Filters: (i) minimum Length Filter (requires >=50 bases), Start Codon Filter (requires start codon at beginning of the gene), Stop Codon Filter (requires single stop codon at end of gene), Homologous Gene Filter (requires no multiple copies of gene with BLAST overlap>=100bp, identity>=90.0%), Gene Overlap Filter (requires no overlap with other genes >4 bases) and Exclude Sequences Filter (requires no BLAST hit with overlap ≥ 100bp and identity ≥ 90.0% with sequences defined to be excluded (e.g. plasmids)). Targets were further filtered using the 96 genomes from the penetration set according to the default SeqSphere+ Query Genome Filters: (i) Blast search (requires BLAST hit with overlap=100%, identity ≥ 90% in every query genome; BLAST v2.2.12 with options: word size=11, mismatch penalty=-1, Match reward=1, Gap open costs=5, Gap extension costs=2) and (ii) Stop Codon Percentage Filter (requires single stop codon at end of gene in >80% penetration genomes). From the initial set of 4,920 loci, 30 were discarded because multiple copies were found in the seed genome (n=28) or because they were located on plasmids (n=2; exclude sequence filter) (Supplementary Data 2 and Supplementary Fig. 3). In total, 1,986 loci were moved to accessory genome because they overlapped in the seed genome (n=338), were not found in each of the query genomes (n=1,646) or because more than 20% of the query genomes had an invalid/missing stop codon for this locus (n=2). The remaining hard defined core genome that makes up the final cgMLST scheme thus consisted of 2,904 loci (Supplementary Data 2).

## 2. Technical validation of Mab cgMLST scheme

To assess the robustness of the scheme, we compared cgMLST results obtained for genomes generated from the same isolate with different assembly approaches (see Methods and Supplementary Data 4). This technical validation was performed using a diverse set of 30 Mab strains (10 strains of each Mab subspecies) for which both publicly available WGS read sets (Illumina fastQ files) and assemblies (fastA files) were available (Supplementary Data 1 and 3 and Supplementary Fig. 1). These isolates were randomly selected from the public

assembly set, originated from seven different countries (USA, Brasil, Denmark, Netherlands, UK, Ireland and Australia) and belonged to DCCs and non-DCC clades. Most isolates (28 out of 30) were not included in the scheme creation set. Assemblies from the short read sets were made using two popular *de novo* (SPAdes and skesa) assemblers and one mapping assembler (BWA-MEM), using different assembly pipelines (SeqSphere$^+$ and shovill) and using different read preprocessing steps (default trimming or prior read error correction turned off or on) (see Methods and Supplementary Data 4). Next to the average percentage of "good" cgMLST targets (i.e. those for which an allele number was assigned), we also recorded the processing time to get an idea about the total time required for cgMLST analysis (Supplementary Data 4).

For each assembly (method), more than 98% good cgMLST targets were identified. However, the number of good targets was significantly lower for the mapping approach compared to *de novo* assembly approaches (Supplementary Data 4) ($p<0.05$). Total processing time per sample ranged between 4.3 and 18.4 minutes (Supplementary Data 4). Important to note is that processing time depends not only on the software tool, but also on your hardware configuration. The actual time to determine the cgMLST profile from the assembly in SeqSphere+ (i.e. searching for all 2,904 cgMLST loci and translation of the sequences into allele numbers) was on average 4.5 minutes per sample (Supplementary Data 4). Once cgMLST profiles were calculated and stored in the SeqSphere+ database, the time required to compare cgMLST profiles (i.e. calculate distances) or create minimum spanning trees was neglectable (<1 min for 210 profiles).

We calculated the cgMLST distance as the number of cgMLST loci with allele differences between the different assemblies, not considering missing alleles in the pairwise comparisons. If different assemblies of the same isolate were analyzed, there were no cgMLST loci with a different allele number (cgMLST distance = 0) between the assemblies generated with shovill and SeqSphere$^+$ (Supplementary Fig. 4).

However, for 16 out of 30 strains, one up to six allele differences were found between publicly available assemblies (i.e. those downloaded from the public repository RefSeq) and those generated as part of this study in shovill or SeqSphere+ (Supplementary Fig. 4). For 25 strains, 1 up to 19 cgMLST targets with different allele numbers were found when the mapping approach was compared with de novo assembly approaches (Supplementary Fig. 4).

Overall, the results indicate that (i) a *de novo* assembly approach is preferred over a mapping approach to create draft assemblies of Mab isolates for subsequent cgMLST analysis (ii) cgMLST results of assemblies generated with different assembly approaches can be compared directly.

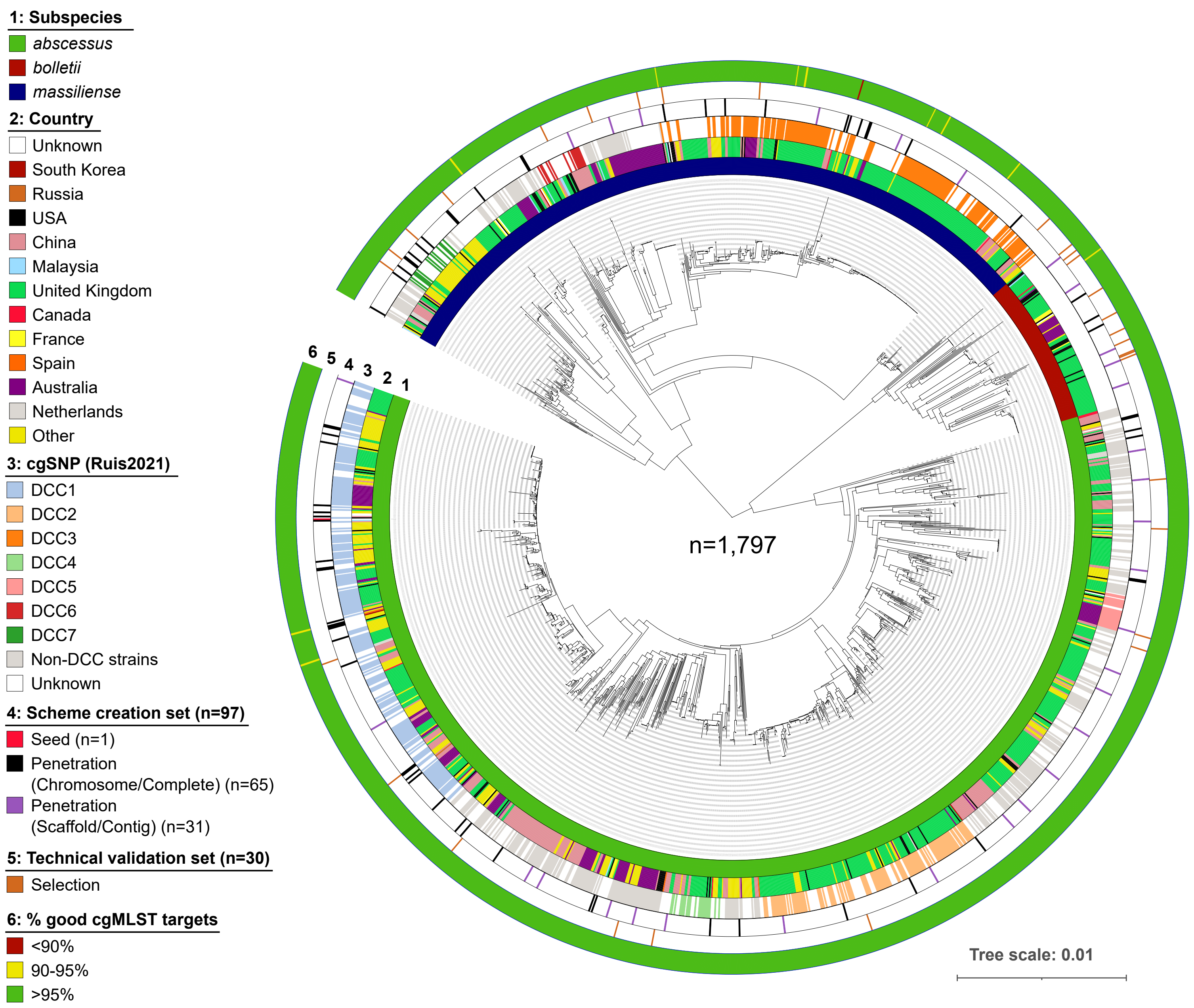


**Supplementary Figure 1: Mash distance-based neighbor-joining tree (NJ) of 1,797 *Mycobacterium abscessus* isolates (public assembly set).** 1. Subspecies classification. For isolates with no classification available, subspecies was derived based on the isolates' position in this tree. 2. Country of isolation 3. DCC classification as reported by Ruis and coworkers. 4. Genomes used for cgMLST scheme creation: seed genome (n=1) and penetration set (n=96) 5. Genomes used for technical validation of the cgMLST scheme (n=30). 6. % good cgMLST targets as defined by SeqSphere+.

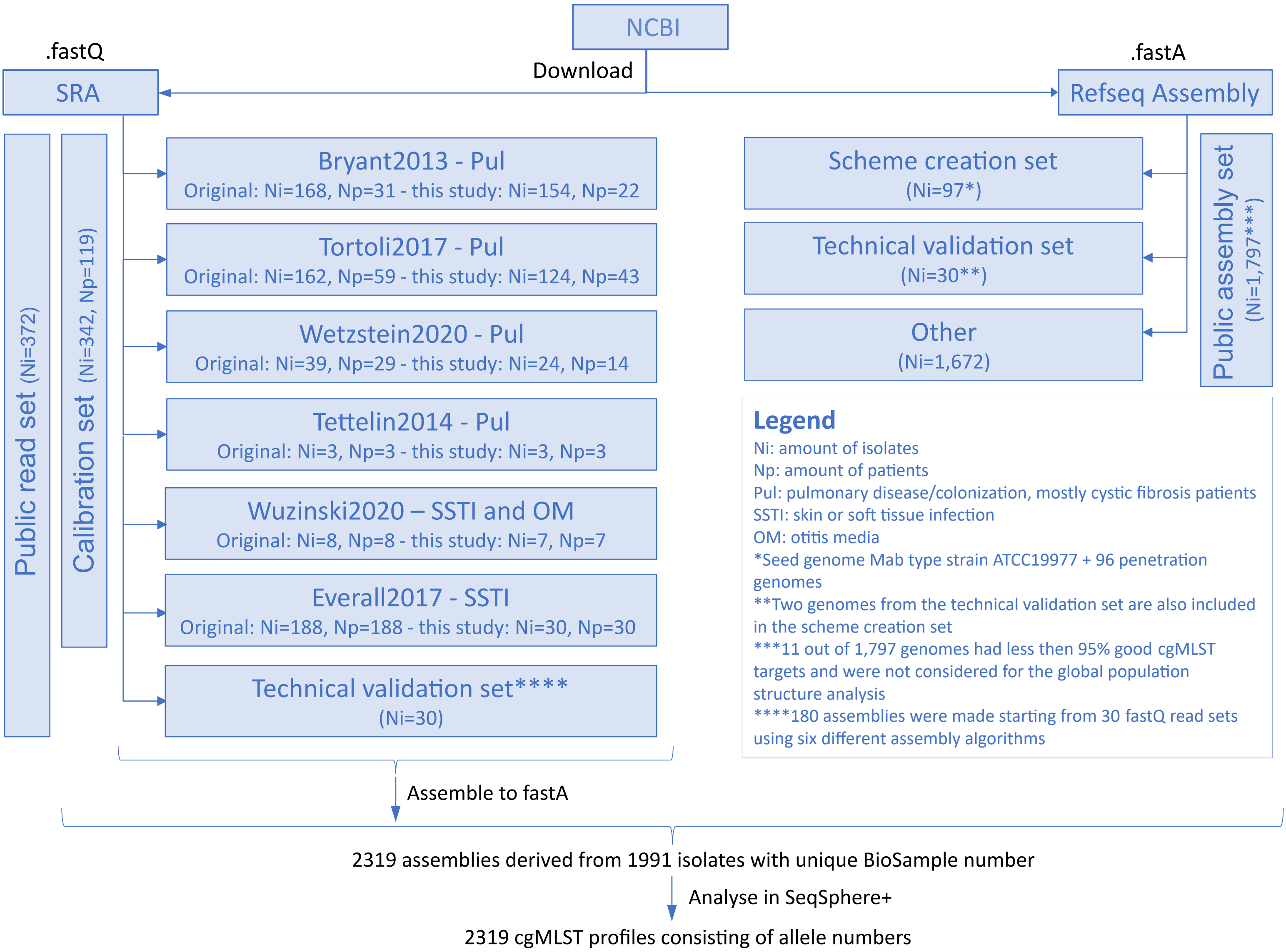


**Supplementary Figure 2: Overview of datasets used for cgMLST scheme creation, validation and threshold calibration.**

**1. Scheme creation**

**1a:** Define Seed Genome (n=1)

→ Initial set of loci: 4,920

**1b:** Define Penetration Genomes (n=96) and genes to exclude (n=17)

→ 30 loci discarded; 1,986 accessory loci

**Resulting cgMLST scheme:**

2,904 core loci (59%)

**Seed genome**: Type strain $Mab_A$ ATCC 19977 (NC_010397.1; Ripoll et al. 2009) 5,067,172 bp; 4,920 coding sequences

**Final Penetration set:** 65 finished assemblies and 31 draft genomes, covering all subspecies and DCCs

**Genes to exclude:** genes from all publicly available plasmids (n=17)

→ *28 targets discarded by „Seed Genome Homologous Gene Filter"*
→ *2 targets discarded by „Seed Genome Exclude Sequences Filter"*
→ *338 targets moved to Accessory by „Seed Genome Gene Overlap Filter"*
→ *1646 targets moved to Accessory by „Query Genome BLAST Search"*
→ *2 targets moved to Accessory by "Penetration Query Genomes Stop Codon Percentage Filter"*

**2. Scheme validation**

**2a.** Technical validation

**2b.** Extended validation and comparison with other genotyping methods

**Technical validation set**: 210 assemblies for 30 isolates (10 $Mab_A$, 10 $Mab_M$, 10 $Mab_B$)

→ *CgMLST profiles for assemblies made using different assembly approaches are highly similar*

**Public assembly set:** 1,797 assemblies (1110 $Mab_A$, 563 $Mab_M$, 124 $Mab_B$)

→ *1,786 out of 1,797 genomes with ≥ 95% good cgMLST targets indicates stable scheme*
→ *Population structure with regard to subspecies and dominant circulating clones (DCC) congruent with cgSNP but DCC3 could be split in DCC3a and DCC3b*
→ *Higher resolution compared to traditional MLST; MLST types correlate with DCCs.*
→ *Most DCC isolates have less than 250 alleles difference compared to a DCC reference genome*

**3. Threshold calibration**

**Calibration set:** 342 isolates from 119 patients (read datasets)

**a) Outbreak/transmission set**: 76 isolates from 76 patients

**b) Sequential isolates set**: 291 isolates from 69 patients

→ *SNP and cgMLST distances in same range*
→ *1620 out of 1637 (99%) pairwise comparisons between epidemiologically linked isolates < 25 alleles*
→ *1484 out of 1637 (90%) pairwise comparisons between epidemiologically linked isolates < 10 alleles*
→ *Initial cluster threshold of 25 (possible epi-link) with <10 alleles indicating probable/recent epi-link*

**Supplementary Figure 3: Workflow used for cgMLST scheme creation, validation and threshold calibration.**

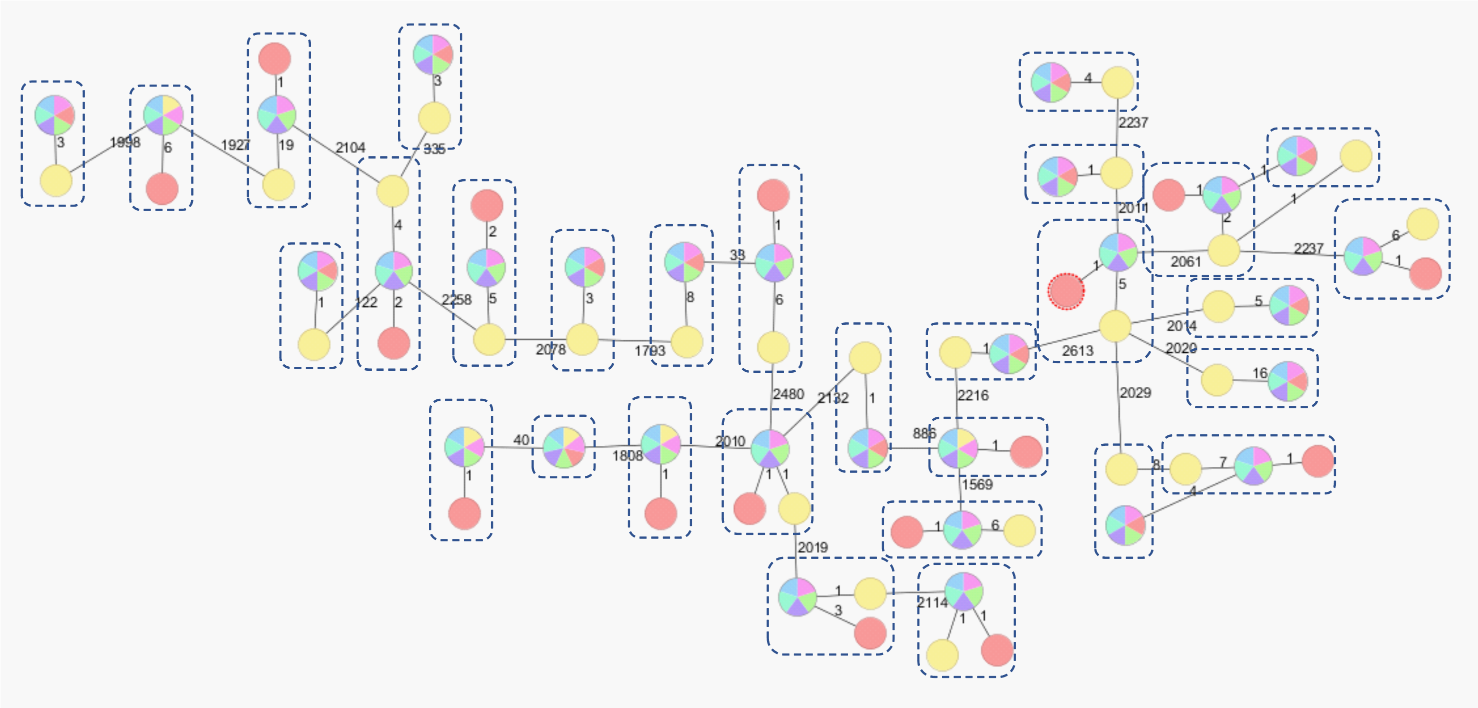


skesa_denovo_shovill_noREC_Trim
skesa_denovo_shovill_REC_Trim
spades_denovo_shovill_noREC_Trim
bwa-mem_mapping_seqpshere_noREC_NoTrim
skesa_denovo_seqsphere_noREC_noTrim
skesa_denovo_seqsphere_noREC_Trim
public_assemblies_refseq

**Supplementary Figure 4: CgMLST-based minimum spanning tree of 30 isolates included in the technical validation set.** For each isolate, cgMLST analysis was performed on seven assemblies generated using different assemblers and/or preprocessing steps (Details in Methods and Supplementary Table 4). REC: read error correction. The number on the branches represents the amount of cgMLST allele differences (distance) between assemblies. Assemblies belonging to the same isolate are grouped with dashed lines. Each node represents an assembly from a particular isolate. Assemblies without any allelic differences are collapsed into one node. For visualisation purposes, the branch length is not proportional to the distance.

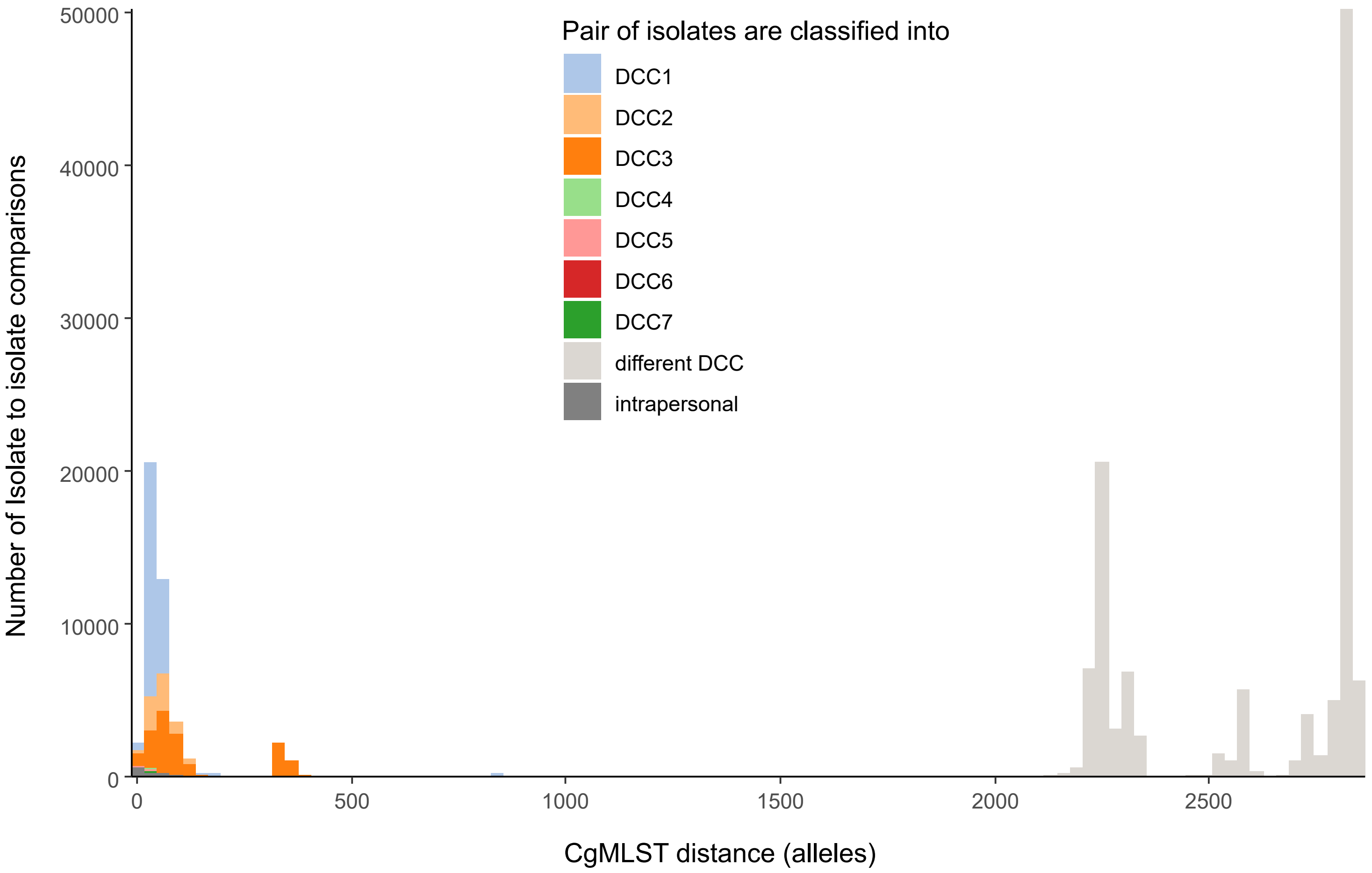


**Supplementary Figure 5: Histogram including all pairwise comparisons between 571 isolates previously classified into one of seven dominant circulating clone (DCC) complexes.**

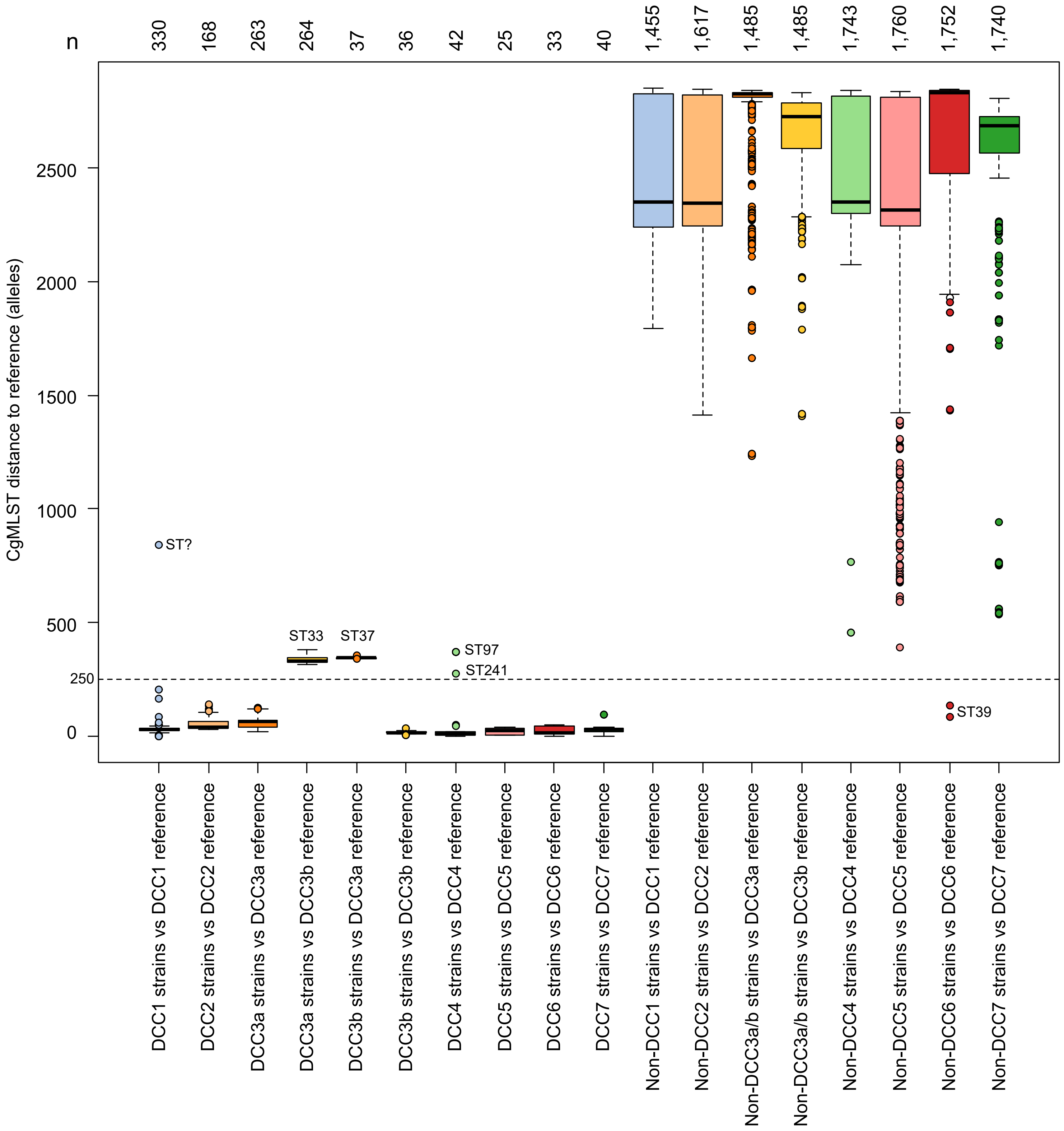


**Supplementary Figure 6: Boxplots of pairwise genetic distances between DCC reference genomes and 1,785 isolates from the global population analysis set.** DCC1 reference: NC_010397.1 (ST5); DCC2 reference: GCF_017189395.1 (ST9); DCC3a reference: GCF_003076795.1 (ST33); DCC3b reference: GCF_014843175.1 (ST37); DCC4 reference: GCF_900134595.1 (ST101), DCC5 reference: GCF_900141545.1 (ST23), DCC6 reference: GCF_002799795.1 (ST39), DCC7 reference: GCF_002140035.1 (ST42). Colors are according to the reference genome. Sample sizes (i.e. amount of pairwise comparisons towards reference genome) are indicated above figure. Solid lines indicate median pairwise allele distance, box represent interquartile range (IQR), whiskers extend to 1.5 of the IQR, dots represent outliers. STs are mentioned only for outliers (i.e. isolates for which classification derived from phylogenetic positioning (see Figure 1) does not correspond to the classification obtained using a threshold of <250 allele differences with reference genome.
ST?: ST could not be determined due to incomplete MLST sequences, therefore this genome might be of bad quality. ST33 and ST37: these outliers encourage the split of DCC3 into DCC3a and DCC3b. ST97 and ST241: as these outliers form a separate monophyletic clade, have more than 250 allele differences with DCC4 reference and have different STs compared to the rest of the DCC4 isolates, one might exclude them from DCC4. ST39: as these outliers have the same ST type as all other DCC6 strains and have less than 250 alleles difference with the DCC6 reference genome, one might want to consider these isolates as DCC6 strains.

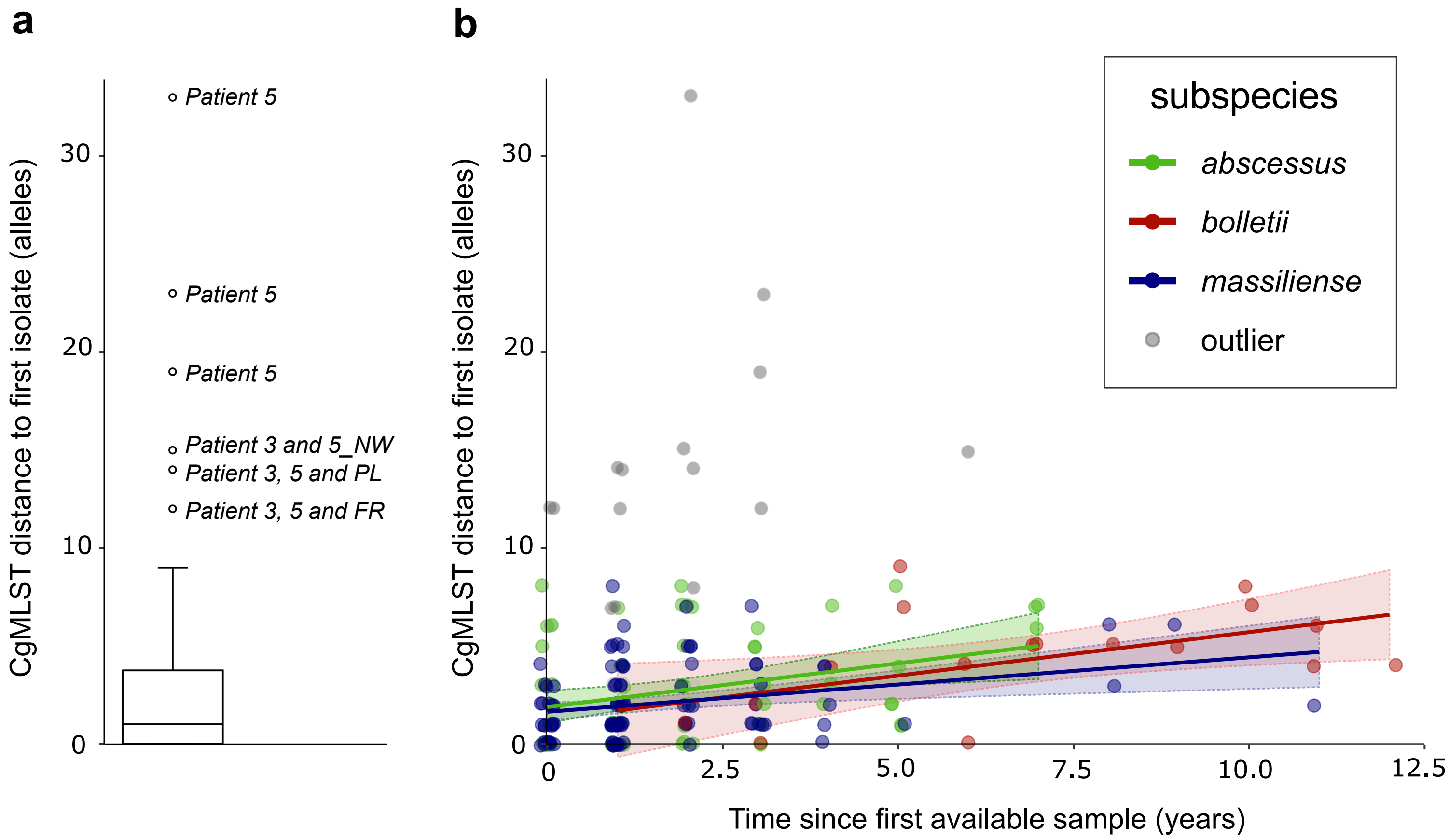


***Supplementary Figure 7: Within-patient diversity.*** Pairwise distances are calculated as the amount of allelic differences (cgMLST analysis) between an isolate (collected at x years after the first available isolate) and the first available isolate. Data is plotted for 43 patients with sequential *Mycobacterium abscessus* subsp. *abscessus* (MabA) isolates, 18 patients with sequential subsp. *massiliense* (MabM) isolates and 8 patients with sequential Mab subsp. *bolletii* (MabB) isolates, from three previous studies (Bryant2013, Tortoli2017 and Wetzstein2020). a. Boxplot to identify outliers with high genetic distance compared to first isolate (n=291 pairwise comparisons). These outliers might be the result of mixed infections with closely related isolates or hypermutator strains. Solid line indicate median pairwise allele distance, box represent interquartile range (IQR), whiskers extend to 1.5 of the IQR, dots represent outliers. b. Correlation of genetic divergence with the duration of infection (scatter plot with jitter function). Linear regressions were fitted by the least-squares method and did not include outliers (i.e. patient 3, 5, PL, FR and 5_NW). The slope of the linear fit was used to estimate evolutionary rate. The estimated evolutionary rate for Mab (including all subspecies but excluding outliers) was 0.38 alleles/genomes/year (95% CI 0.26 – 0.50, t Stat = 6.2, df = 189, p = 2.96E-09, $R2$ = 0.170; regression line not shown) with 0.45 alleles/genome/year (95% CI 0.13-0.76, t Stat = 2.8, df = 68, p = 6.32E-03, R2 = 0.105) for MabA, 0.28 alleles/genome/year (95% CI 0.09-0.46, t Stat = 2.9, df = 101, p = 3.99E-03, R2 = 0.079) for MabM, and 0.44 alleles/genome/year (95% CI 0.08-0.81, t Stat =

## Supplementary references

1. Ruis, C. *et al.* Dissemination of Mycobacterium abscessus via global transmission networks. *Nat. Microbiol. 2021 610* **6**, 1279–1288 (2021)
