# Supplementary material for: Delineating Mycobacterium abscessus population structure and transmission employing high-resolution core genome multilocus sequence typing
Source: Nat Commun. 2022 Aug 23;13:4936. doi: 10.1038/s41467-022-32122-5 (PMC9399081; doi:10.1038/s41467-022-32122-5)
Supplement: Supplementary file 3 — Description of Additional Supplementary Files [file 41467_2022_32122_MOESM3_ESM.pdf]

Description of Additional Supplementary Files

File Name: Supplementary Data 1
Description: Accession numbers and metadata of all publicly available assemblies (fastA) used in this study (n=1,797; public assembly set). This set includes the technical validation set (n=30), scheme creation set (n=97) and population structure analysis set (n=1,786). #N/A: Not assigned. 1 Subspecies was initially determined using phylogenetic placement in mashtree containing isolates with known classification. 2 isolates with unknown DCC status that were positioned in the NJ tree within a clade containing isolates with known DCC status were classified into the corresponding DCC (see Figure 1). 3 For references used, see Supplementary Data 5.

File Name: Supplementary Data 2
Description: Loci from *Mycobacterium abscessus* type strain ATCC19977 included and excluded from the cgMLST scheme.

File Name: Supplementary Data 3
Description: Accession numbers and metadata of all publicly available read data sets (fastQ) used in this study (n=372; public read set). This set includes the technical validation set (n=30) and calibration set (n=342). The calibration set includes sequential isolates from cystic fibrosis (CF) patients (sequential isolate set) as well as isolates from well-defined outbreaks or putative transmission events (outbreak/transmission set). #N/A: not assigned.

File Name: Supplementary Data 4
Description: Assembly approaches used for the technical validation set to assess robustness of the cgMLST scheme. Reported processing times include assembly or mapping with shovill or SeqSphere+ and extraction of the cgMLST profile in SeqSphere+. It does not include the time to compare different cgMLST profiles (e.g. to generate minimum spanning trees), which is neglectable anyway. Values are averaged across 30 isolates from the technical validation set.

File Name: Supplementary Data 5
Description: *Mycobacterium abscessus* type and reference strains that can be used for subspecies and dominant circulating clone (DCC) classification.

File Name: Supplementary Data 6
Description: Comparison of the genetic cgMLST (based on 2904 loci) and traditional MLST (7 loci) distances between 571 previously identified DCC strains (Ruis et al. 2021; part of public assembly set). The most prevalent ST types per DCC are highlighted in orange. Loci with a different allele number compared to the most prevalent ST are highlighted in blue. Samples with less than 95% good cgMLST targets or partial/ambiguous MLST loci were excluded. *ST types are defined using the nomenclature from pubMLST. **ST could not be determined due to loci with ambiguous bases or truncated loci and were therefore excluded from max. pairwise MLST distance calculation. ***Pairwise comparisons between isolates from same patient were excluded.

File Name: Supplementary Data 7

Description: Overview of outbreak and putative transmission clusters used in the meta-analysis. *: In the original study (Tortoli2017), IR and GI isolates are within this cluster, but not connected with less than 25 SNPs to the other cluster members

File Name: Supplementary Data 8

Description: Genomic regions with SNPs between GI2, IR1, MC1 and TE1. Consecutive genes with a high density of SNPs are indicated in bold. 1 With default settings of the MTBseq pipeline, variant positions within 12 bp in same isolate are excluded to correct for mapping artefacts around indels or rearrangements

File Name: Supplementary Data 9

Description: Average and maximum pairwise distances between sequential isolates from the same patient. Distances were computed for 291 isolates from 69 patients (Sequential isolates set).
